# Analysis of the Quality of Selected Vegetarian Products Available on the Polish Market Compared to Their Homemade Equivalents

**DOI:** 10.3390/foods14050891

**Published:** 2025-03-05

**Authors:** Agata Kiciak, Natalia Kuczka, Renata Barczyńska, Wiktoria Staśkiewicz-Bartecka, Agnieszka Białek-Dratwa, Anna-Maria Sapała, Oskar Kowalski, Marek Kardas

**Affiliations:** 1 Department of Food Technology and Quality Assessment, School of Public Health in Bytom, Medical University of Silesia in Katowice, ul. Jordana 19, 41-808 Zabrze, Poland; natalia.k4940@gmail.com (N.K.); wstaskiewicz@sum.edu.pl (W.S.-B.); mkardas@sum.edu.pl (M.K.); 2 Department of Dietetics and Food Science, Faculty of Science, Natural and Technical Sciences, Jan Długosz University in Częstochowa, Aleja Armii Krajowej 13/15, 42-200 Czestochowa, Poland; r.barczynska-felusiak@ujd.edu.pl (R.B.); am.stelmach@ujd.edu.pl (A.-M.S.); 3 Department Human Nutrition, Department of Dietetics, School of Public Health in Bytom, Medical University of Silesia in Katowice, ul. Jordana 19, 41-808 Zabrze, Poland; abialek@sum.edu.pl (A.B.-D.); okowalski@sum.edu.pl (O.K.)

**Keywords:** plant-based products, plant-based analogs, vegetarian diet

## Abstract

Plant-based products are gaining increasing popularity, making a vegetarian diet a fundamental part of nutrition among many social groups. The aim of this study was to assess the quality of selected vegetarian products available on the Polish market and their homemade counterparts. Additionally, consumer preferences and dietary behaviors regarding vegetarian diets and products available on the Polish market were analyzed. The consumer evaluation of the intensity of selected sensory attributes using the five-point scale method showed that, among the hummus samples, the natural hummus received the highest rating among all the tested products. In the falafel group, the homemade falafel received the highest scores. The consumer preference assessment using the ranking method, which considered the taste of the products, indicated that traditional hummus received the highest scores. In the falafel group, the highest number of points was awarded to the homemade falafel and the chickpea–spelt falafel. The majority of respondents declared that the taste of the tested products was a very important quality determinant. The choice of plant-based products made by consumers primarily depends on individual dietary preferences. The key determinant influencing consumers when selecting plant-based products is taste, which plays a crucial role in their decision to repurchase.

## 1. Introduction

In recent years, a growing body of scientific evidence has suggested that following a healthy and balanced plant-based diet provides significant health benefits compared to a diet rich in meat and other animal-derived products [1].

Vegetarianism has become an increasingly popular lifestyle choice among a growing portion of society. While some vegetarians choose to use dietary supplements to address potential nutrient gaps, a well-planned and balanced vegetarian diet can provide the essential nutrients and minerals required for maintaining health and proper bodily functions [2].

Currently, diets based on plant-based products are becoming increasingly popular worldwide, including in Poland. It is estimated that one of the largest vegetarian communities is found in India, comprising as much as 35.71% of the entire population. European countries such as Italy (9.67%), Germany (9.02%), and the United Kingdom (8.57%) also have a high percentage of individuals following plant-based diets [1,3].

The plant-based product market, responding to the increasing consumer demand for plant-based food, is introducing a significantly larger variety of new products to Poland, catering to individuals following vegan or vegetarian diets [4,5,6]. The growing popularity of plant-based products is driven by the significant rise in consumer awareness regarding the negative effects of traditional diets that include animal-based products.

In recent years, much more attention has been given to food that is both safe and healthy for consumers. An active lifestyle, combined with proper nutrition, plays a key role in the functioning of every individual. Acquiring knowledge about healthy eating and the impact of consumed food on the body brings numerous benefits. Applying appropriate dietary techniques in daily life improves health and overall well-being [2].

The trend surrounding vegetarian food has gained significantly more visibility in global markets in recent years. Vegetarian diets are also becoming increasingly popular in Poland, earning greater recognition within society. Despite the growing interest in vegetarian diets, the definition of this diet remains unclear [7,8].

The term “vegetarianism” is derived from two words: vegetabilis (meaning plant-based) and vegetare (meaning to grow, thrive, or develop). A vegetarian diet is essentially a plant-based diet aimed at eliminating or limiting animal-based products, such as meat from slaughtered animals, game, poultry, and sometimes fish. The concept of vegetarianism is often associated with “plant-based eating” or “a plant-based diet”, but it does not necessarily imply the complete exclusion of animal-derived products, which contributes to the lack of a clear definition of vegetarianism [9].

A common term used in the literature is “plant-based diet”, which encompasses both vegetarian and vegan diets. These diets predominantly rely on plant-based analogs [7,9].

Foods classified as vegetarian are primarily based on plant-derived products. Vegetarianism emphasizes the consumption of foods such as fruits, vegetables, whole grains, legumes, nuts, seeds, cereal crops, oilseeds, root vegetables, and mushrooms, while aiming to eliminate or reduce the intake of meat and animal-derived products.

According to the International Vegetarian Union (IVU), vegetarianism is defined as: “a diet based on plant-derived foods, with or without the addition of dairy products, eggs, and/or honey” [10].

The lack of consensus regarding the proper definition of a vegetarian diet and a plant-based diet can lead to misunderstandings and misinterpretation of the terminology used. Despite the well-documented health benefits of a vegetarian diet, its impact on the quality of life of individuals following it still requires thorough investigation [8].

Experts from the World Health Organization (WHO) emphasize that changes in dietary patterns can have either a positive or negative impact on the quality of life of societies. Proper nutrition ensures the supply of essential nutrients and minerals necessary for the body’s proper functioning. Considering the growing number of people adopting vegetarian diets and the increasing interest in the topic in recent years, it is crucial to gain a proper understanding of vegetarianism and its influence on the quality of life [8,11,12].

Vegetarian products are widely available in grocery and organic stores. Large supermarkets and discount chains are introducing them to expand their offerings, and their sales are systematically increasing [3,9].

Among the many variations of diets that eliminate animal-derived products, vegetarian diets can be categorized based on the range of consumed products. The most common types include veganism, lacto-vegetarianism, raw foodism, fruitarianism, semi-vegetarianism, pollo-vegetarianism, flexitarianism, and ovo-vegetarianism [1,13].

### General Characteristics of Selected Vegetarian Products Considering Their Nutritional Value

The legume family consists of plants characterized by the production of pods containing seeds inside. Common edible legumes include chickpeas, lentils, soybeans, lupins, green beans, alfalfa, peas, broad beans, dry peas, and dry beans [14]. These edible seeds provide a balanced source of plant-based protein while enriching daily diets with dietary fiber, nutrients, and micro- and macroelements [15].

Chickpeas are among the oldest and most widely consumed legumes, originating from the Middle East. They are characterized by a delicate, slightly nutty flavor and a low content of sulfur-containing amino acids (cysteine and methionine). Dried chickpeas have a low fat content, approximately 6.0 g per 100 g of dried seeds, but contain as much as 20 g of protein per 100 g of dried seeds [16].

Tahini, also known as sesame paste, is made from roasted, ground sesame seeds. A key component of hummus paste, tahini is rich in unsaturated fatty acids, tocopherols, and antioxidant lignans. It also contains essential minerals such as calcium and phosphorus [17].

Like chickpeas, sesame owes its health-promoting properties to its nutrient content, including dietary fiber, protein, calcium, and vitamin B3 (niacin), which provide significant health benefits for the body. Additionally, niacin plays an important role in metabolic processes, the synthesis and breakdown of fatty acids, and regulating blood cholesterol levels by increasing HDL cholesterol. Among the nutrients found in sesame, thiamine ranks second, supporting the proper functioning of the nervous system and preventing concentration disorders and depression [16,18,19].

Vegetarian diets and plant-based products are on the rise, yet there is a lack of comparative analysis between commercially available vegetarian products and their homemade counterparts in Poland. To our knowledge, this is the first study to systematically compare the sensory quality and consumer perception of these two categories. By filling this gap, our study provides insight into the quality attributes that influence consumer preferences and decision making, which can help both producers and consumers make informed choices.

The main objective of the study was to assess the quality of selected vegetarian products available on the Polish market and their homemade equivalents.

The following specific objectives were established to achieve the main goal: consumer evaluation of the intensity of sensory characteristics using a five-point scale method, consumer evaluation of the sensory attractiveness of vegetarian products and their homemade equivalents using a ranking method, consumer assessment of the quality determinants of vegetarian products conducted through a custom-designed questionnaire, and consumer evaluation characterizing the preferences and dietary behaviors regarding vegetarian diets and products available on the Polish market.

## 2. Materials and Methods

### 2.1. Sensory Experiment Design

The conducted study was experimental in nature due to the significant contribution of independent work, which involved the preparation of homemade equivalents and the development of appropriate recipes for selected vegetarian products. The process of selecting suitable ingredient proportions and components used in product preparation was meticulous and time consuming. A precisely developed preparation technology and multiple attempts to create comparable vegetarian products to those available commercially contributed to obtaining homemade equivalents of products available on the Polish market.

The research material consisted of two groups of selected vegetarian products and their homemade equivalents. The study focused on hummus paste and falafel.

**Figure A1:**
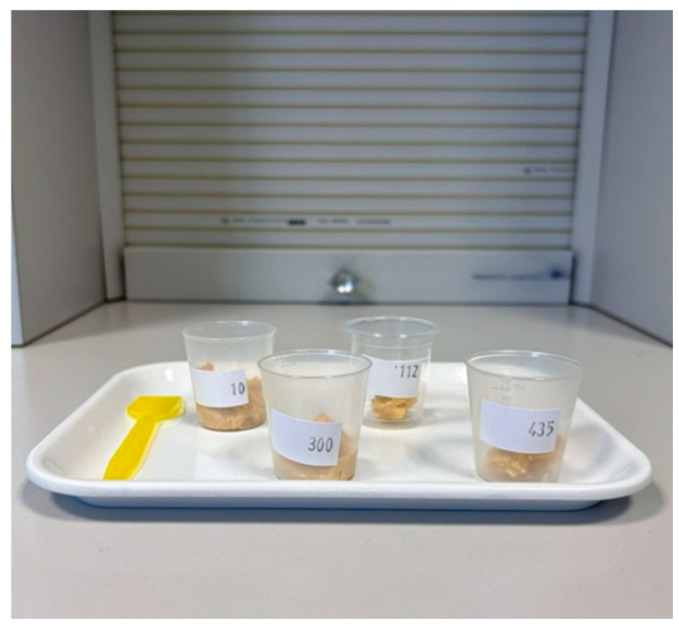
Prepared research material representing hummus and its author’s equivalent. Source: author’s photograph.

**Figure A2:**
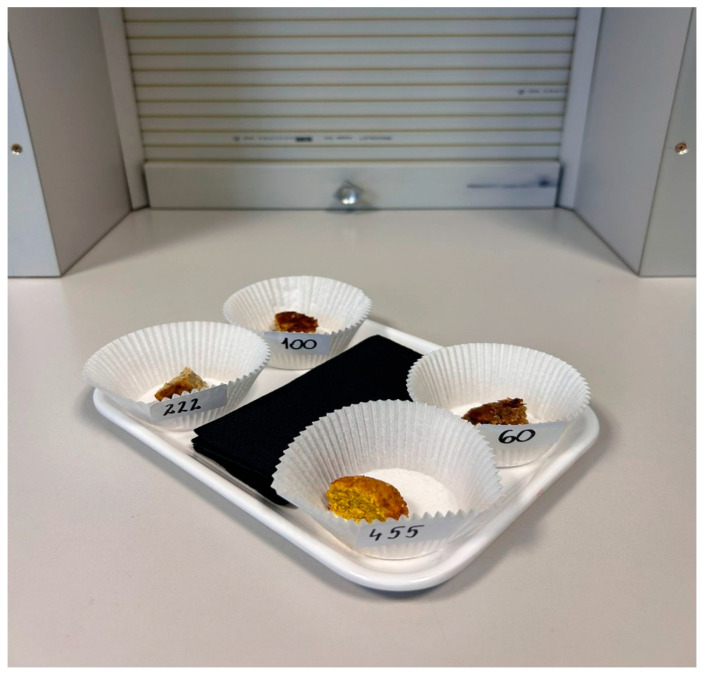
Prepared test material representing falafel and the author’s counterpart. Source: author’s photograph.

The preparation of samples for the evaluation of vegetarian products and their proprietary counterparts is shown in Table A1 and Figure A1 and Figure A2.

The homemade equivalents were developed based on nutritional benchmarking, ensuring comparability with commercially available products. The formulation process involved the following: ingredient selection—ingredients were chosen based on the typical composition of commercial products while adhering to traditional recipes; expert consultation—a team of food technologists and dietitians provided guidance to ensure that the recipes maintained nutritional balance and sensory characteristics similar to market-available products; iterative testing—multiple test batches were prepared and refined based on sensory evaluations to match commercial equivalents in terms of texture, taste, and appearance; nutritional benchmarking—the macronutrient composition of homemade products was analyzed and compared to their commercial counterparts to confirm their similarity.

The selected vegetarian products and their homemade equivalents used for sensory evaluation varied in composition depending on the manufacturer [20,21]. A detailed analysis of the ingredients contained in both the vegetarian products and their homemade counterparts is presented in Table 1.

The study was conducted in the Sensory Analysis Laboratory of the Department of Dietetics at the Medical University of Silesia in Katowice, Faculty of Public Health in Bytom. The laboratory where the study was carried out met the requirements and guidelines of the PN-EN ISO 8589:2010 standard for Sensory Analysis—General Guidelines for the Design of Sensory Analysis Laboratories [22]. The study was conducted in accordance with the Declaration of Helsinki of the World Medical Association. The study protocol (KNW-0022/KB1/73/I/16) was reviewed and approved by the Bioethics Committee of the Medical University of Silesia in Katowice. Each participant provided informed consent to participate in the study and was informed about the anonymity of the results.

Vegetarian products and the ingredients necessary for preparing the proprietary versions of the selected food items were purchased from popular supermarkets such as Kaufland and Biedronka. Additionally, online stores, including Organic Market, Urban Vegan, Bio Sklep, and the shopping platform, Allegro, were used. The products were regularly purchased before each study session, starting in 2023.

In the analytical part of the study, the sensory evaluation of the products was carried out in two stages. In the first stage, 126 undergraduate and graduate students of Dietetics at the Medical University of Silesia in Katowice were divided into subgroups of 5–6 participants. They assessed the products using a five-point scale, which was treated as a consumer test method rather than a rigorous expert panel assessment. In the second stage, the second research group consisted of high school students participating in educational and developmental activities as part of the “Appetite for Healthy Eating” project, co-funded by the Ministry of Education and Science under the “Social Responsibility of Science” program, implemented at the Department of Dietetics in Zabrze-Rokitnica. In this part of the study, the ranking method was applied.

A properly equipped laboratory was used for conducting the sensory analysis of the selected food products. The study was carried out from September 2023 to March 2024.

During the study, the intensity of the selected functional characteristics, such as color, aroma, consistency, appearance, and taste, was evaluated. The order of the analyzed attributes was not random. At the beginning of the study, the focus was on visually assessed characteristics, namely appearance, color, aroma, and consistency, while taste evaluation was conducted at the end.

### 2.2. Evaluation of Vegetarian Products Using the Five-Point Method

A proprietary five-point rating scale was used for the assessment (5—very good product quality, 1—disqualifying quality of the tested product). The evaluation was based on Polish Standards PN-ISO 22935-1 Sensory Analysis, Part 1: General Guidelines for Recruitment, Selection, Training, and Monitoring of Assessors [23].

The detailed procedure for preparing the vegetarian products and their proprietary equivalents for evaluation is presented in Appendix A.

Each participant in the study received a set of four coded samples in two separate sessions. Each sample in a given set had a specific weight and belonged to a selected group of food products. Additionally, the respondents were provided with a bottle of still mineral water. In the first stage, hummus paste was served, followed by falafel.

Furthermore, the assessors received evaluation cards listing the quality indicators for all the tested attributes, along with a sensory evaluation sheet for assessing the samples. For each evaluated characteristic, a weighting coefficient was determined, which was then used to multiply the numerical values assigned by the respondents.

The intensity of the evaluated attributes was assessed by 126 students from the Dietetics program (both undergraduate and graduate) at the Medical University of Silesia in Katowice, including 103 women (81.8%) and 23 men (18.3%). The selected five-point scale method was applied due to its appropriate level of complexity, which was tailored to the skills and experience of the student group participating in the study.

### 2.3. Evaluation of Vegetarian Products Using the Ranking Method

Another component of the study was the sensory evaluation of selected vegetarian products using the ranking method. Two attributes of plant-based products were assessed: appearance and taste. The participants were tasked with assigning each sample a corresponding rank (number 1—least desirable attribute, number 4—most desirable attribute).

A total of 131 high school students participating in the “Appetite for Healthy Eating” project, co-financed by the Ministry of Education and Science under the “Social Responsibility of Science” program, took part in the evaluation. The study group consisted of 81 girls (61.8%) and 50 boys (38.2%).

Each participant received a set of four coded samples in two separate sessions. Each sample in a given set had a specific weight and belonged to a selected group of food products. Additionally, the respondents were provided with a bottle of still mineral water. In the first stage, hummus paste was served, followed by falafel.

The ranking method was chosen due to its low level of complexity, making it well-suited to the skills and experience of the “Appetite for Healthy Eating” project participants. High school students could easily understand and accurately conduct the sensory analysis, ensuring reliable and more precise study results.

### 2.4. Assessment of Consumer Preferences and Eating Behaviour

The third part of the study focused on evaluating preferences and dietary behaviors related to the consumption of selected vegetarian products available on the Polish market. The study was conducted among Dietetics students from the Medical University of Silesia in Katowice and high school students participating in educational and developmental activities within the “Appetite for Healthy Eating” project.

For this purpose, a proprietary survey questionnaire was developed, consisting of two parts. The first part included questions aimed at characterizing the surveyed group in terms of gender, age, place of residence, and type of household. The second part contained 10 closed-ended, single-choice questions assessing consumer preferences and dietary behaviors regarding the consumption of vegetarian products analyzed in the study.

The questionnaire was completed by 126 students (103 women, 23 men) and 131 participants from the “Appetite for Healthy Eating” project (81 girls, 50 boys). In total, the proprietary questionnaire was filled out by 257 respondents (184 women, 73 men).

The prepared survey questionnaire ensured the anonymity of the respondents, who were informed about the purpose of the study and its methodology, and provided their consent to participate.

An additional component of the study involved the characterization of the nutritional value of hummus paste and falafel. The results of this part of the study are presented in the first section of the paper.

### 2.5. Statistical Analysis

All the obtained results were cataloged and analyzed using Microsoft 365 Excel 2024 and Statistica software v.2013, by StatSoft Polska (Krakow, Poland). The χ^2^ test was used to analyze the relationships between the variables, particularly the individual questions from the second part of the survey regarding the dietary preferences of the selected consumer group.

The results for the intensity of the evaluated food product attributes are presented as mean ($\bar{x}$), standard deviation (SD), median (Me), lower (first) quartile (K_0.25_), upper (third) quartile (K_0.75_), minimum (xmin), and maximum (xmax).

To assess the statistical differences for each sensory attribute between the products, a Kruskal–Wallis test was performed, followed by a post-hoc test for comparison between groups.

The significance level was set at *p* < 0.05.

## 3. Results

### 3.1. Nutritional Value of Selected Hummus Pastes

Based on the analysis of the results presented in the table above, it was found that the highest energy value (303.5 kcal per 100 g of product) was observed in homemade hummus (proprietary hummus paste), while the lowest energy value was recorded for traditional hummus (189 kcal per 100 g of product). The fat content, including saturated fatty acids, in the proprietary hummus paste (27.4 g of fat/2.33 g of saturated fatty acids per 100 g of product) and in the natural, organic, gluten-free, bio hummus (24.9 g of fat/3.3 g of saturated fatty acids per 100 g of product) was at a similar level.

Among the selected types of hummus paste, the highest protein content was recorded in natural hummus (8.5 g per 100 g of product). The organic hummus available in a jar is a vegan snack option with a clean ingredient list and organic components. Its recipe includes up to 61% cooked organic chickpeas, organic sunflower oil, organic sesame paste, sea salt, organic apple cider vinegar, and organic dried garlic.

Plant-based products often serve as meat substitutes and are commonly enriched with nutrients to approximate the nutritional value of animal-based products. Individuals who avoid animal-derived products for health, ethical, religious, or humanitarian reasons should pay particular attention to the nutrient content of the foods that they consume [2,19].

Key nutrients of particular importance include protein and dietary fiber, which, when consumed in appropriate amounts, help maintain a well-balanced diet. Among the hummus varieties compared in Table 2, the highest protein content (8.5 g per 100 g of product) was found in natural hummus, while traditional hummus had the lowest protein content (4.7 g per 100 g of product).

### 3.2. Nutritional Value of Selected Falafels

Based on the analysis of the results presented in the table above, it was found that the proprietary homemade falafel had the highest energy value (419.4 kcal per 100 g of product), while the vegan falafel had the lowest energy value (203.0 kcal per 100 g of product). Its composition included soaked chickpeas (63.0%), water, onion, sunflower oil, parsley, salt, and thickening agents such as guar gum, along with spices including garlic, coriander seeds, cumin, and pepper [20]. The product was characterized by a simple, clear, and understandable ingredient list.

The proprietary falafel had a high fat content (19.7 g per 100 g of product) compared to falafel with chickpeas and spelt, which contained only 3.9 g of fat per 100 g of product. The saturated fatty acid content across all four types of falafel remained at a similar level.

Among the selected types of falafel, falafel with chickpeas and spelt had the highest carbohydrate and dietary fiber content, containing 54.0 g of carbohydrates and 13.0 g of fiber per 100 g of product. The ingredients listed on the product label included the following: chickpea flour (41.0%), thermally processed spelt flour (39.0%), dried onion, dried parsley, and spices such as salt, garlic, chili, ginger, coriander, cinnamon, and cumin [21,24].

It has been observed that an adequate protein intake in plant-based diets meets the nutritional requirements for this nutrient through the consumption of diverse and well-balanced foods. The regular consumption of legumes and soy-rich products ensures an adequate protein supply for vegetarians and vegans [24,25,26]. The highest plant protein content was found in homemade falafel and falafel with chickpeas and spelt, both containing 17.0 g of protein per 100 g of product. The data is presented in Table 3.

### 3.3. Preferences and Dietary Behavior Concerning the Consumption of Vegetarian Products Available on the Polish Market

The study included 257 respondents. The participants were divided into three age groups: 13–18 years, 18–24 years, and 24–40 years. Among all the respondents, the largest group (N = 130; 50.6%) belonged to the 13–18 age category. Most of the respondents reported having a primary education (N = 131; 51.0%). The vast majority of the participants resided in urban areas (N = 204; 70.4%), while 230 respondents (89.5%) lived in a family household. A significant majority of the respondents declared that they did not follow a vegetarian diet (N = 236; 91.8%). Only 21 respondents (8.2%) reported following a vegetarian diet. A significant correlation was found between gender and adherence to a vegetarian diet (*p* = 0.0001). A vegetarian diet was more common among women (11.4%) compared to men.

**Figure 1:**
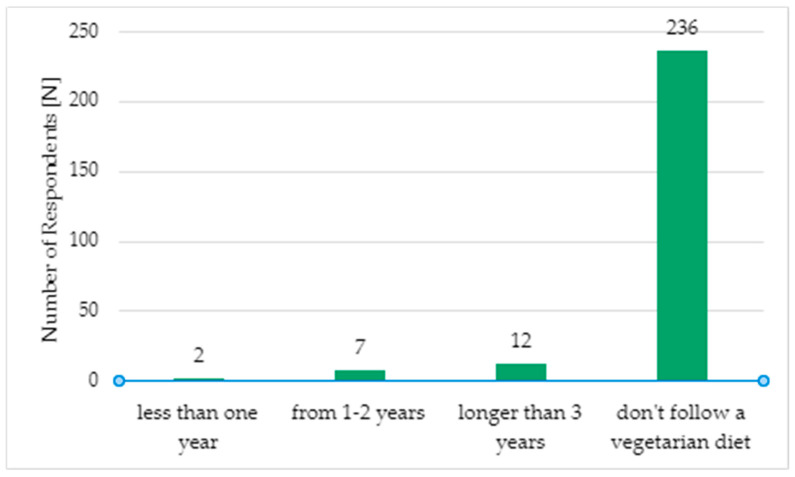
Duration of following a vegetarian diet (N = 257).

To the question “How long have you been following a vegetarian diet?”, 236 respondents (91.48%) answered “I do not follow a vegetarian diet”. The second-most frequently chosen response was “more than 3 years”, selected by 12 respondents (4.7%). Only two people (0.8%) stated that they had been following a vegetarian diet for less than a year. The results are illustrated in Figure 1.

The vast majority of the respondents (N = 75; 29.2%) declared that they had no opinion on the most common drawbacks of a vegetarian diet. However, 70 people (27.7%) identified “the risk of vitamin and nutrient deficiencies” as a primary concern. Forty-one respondents (16.0%) pointed to the insufficient protein intake in the diet, while 34 respondents (13.2%) stated that they did not perceive any disadvantages of a vegetarian diet.

Price and the availability of vegetarian products in stores were not considered significant issues by the respondents, with 26 people (10.1%) mentioning cost and 11 people (4.3%) noting accessibility challenges.

**Figure 2:**
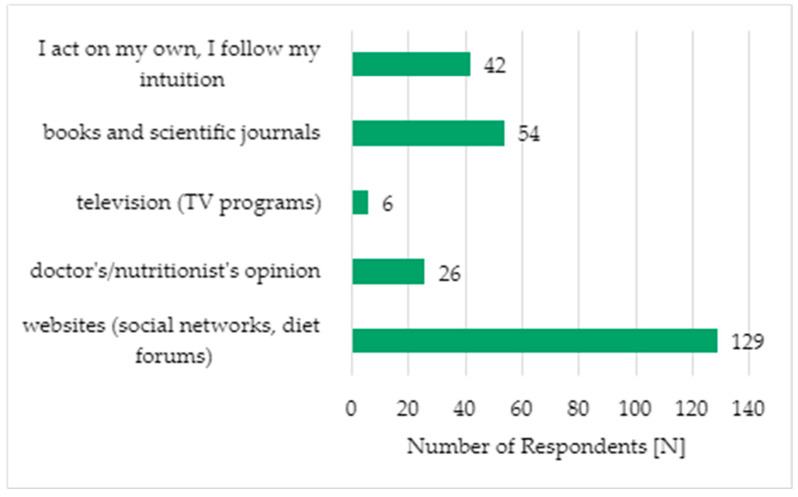
Sources of knowledge about the vegetarian diet (N = 257).

Regarding the sources of knowledge about vegetarian diets, the majority of the respondents acquired information from websites, such as social media platforms and dietary forums (N = 129; 50.2%). The results are illustrated in Figure 2.

A significant correlation was found between the evaluation of the sources of knowledge about the vegetarian diet and age groups. It was observed that the 13–18 age group most frequently acquires information from websites, such as social media platforms and dietary forums, whereas the oldest age group (24–40 years) prefers books and scientific journals (*p* = 0.001). The detailed data are presented in Table 4.

To the question, “Do you believe that following a vegetarian diet in children and adolescents is healthy/appropriate?”, 124 respondents (48.3%) answered “No”. In contrast, only 50 respondents (19.5%) answered “Yes”. Significant differences were found between the groups. Those with a tertiary education were more likely to give the answer that following a vegetarian diet in children and adolescents was healthy. Of those with a tertiary education, 42.6% gave this response compared to 9.9% with a primary education and 19.4% with a secondary education (*p* = 0.001). The data are presented in Table 5.

The largest group of respondents [N = 166; 64.6%] declared that they do not purchase vegetarian products. Meanwhile, 90 respondents (35.0%) stated that they buy vegetarian products in supermarkets, such as Biedronka, Lidl, and Auchan.

To the question, “What factors influence respondents when choosing food products?”, 122 respondents (47.5%) stated that the price of purchased products is a significant determinant. Among the respondents, 111 people (43.2%) declared that the brand is a moderate quality indicator. Additionally, 106 respondents (41.3%) stated that the packaging size is a moderate factor in their purchasing decisions. The composition of food products was an important factor for 109 respondents (42.4%) participating in the study. Taste had a very significant influence on consumer choices, as indicated by 186 respondents (72.4%). Additionally, the expiration date was considered an important quality indicator by 98 participants (38.1%). All the obtained results are presented in Table 6.

The obtained results of the quality indicators’ evaluation for the selected food products showed that taste is a very significant determinant when making purchasing decisions (Me = 4.0). The data are presented in Table 7.

### 3.4. Results of the Sensory Evaluation of Vegetarian Products Using the Five-Point Scale Method

The results of the sensory evaluation using the five-point scale method showed that the natural hummus received the highest ratings from the participants (Me = 4.0). Among all the tested food products, the natural, gluten-free, organic hummus received the lowest score from the respondents (Me = 3.3). The Kruskal–Wallis test revealed statistically significant differences between product groups for color (*p* = 0.017), odor (*p* < 0.001), consistency (*p* < 0.001), and appearance (*p* < 0.001), whereas the differences in taste ratings were not statistically significant (*p* = 0.153). The data are presented in Table 8.

The results of the sensory evaluation using the five-point scale method showed that the proprietary falafel received the highest ratings from the participants (Me = 4.2). Among all the tested food products, the vegan falafel received the lowest score from the respondents (Me = 3.4). The Kruskal–Wallis test revealed statistically significant differences between product groups for color (*p* < 0.001), odor (*p* < 0.001), consistency (*p* < 0.001), appearance (*p* < 0.001), and taste (*p* < 0.001). The detailed data are presented in Table 9.

### 3.5. Results of the Sensory Evaluation of Vegetarian Products Using the Ranking Method

The results of the sensory evaluation using the ranking method showed that the participants assessing the appearance of the tested hummus products (natural, gluten-free, organic hummus; traditional hummus; proprietary hummus) and falafel products (traditional, bio falafel; vegan falafel; chickpea–spelt falafel; proprietary falafel) provided significantly different ratings (*p* < 0.001 for the hummus group; *p* < 0.001 for the falafel group). Specifically, natural hummus and traditional hummus received the highest median scores (Me = 3.0), whereas gluten-free, organic hummus and proprietary hummus were rated lower (Me = 2.0). In the falafel group, the proprietary falafel obtained the highest median score (Me = 3.0), while the remaining products were rated lower (Me = 2.0). The detailed data, including *p*-values and effect sizes, are presented in Table 10.

The results of the sensory evaluation using the ranking method showed statistically significant differences in taste ratings for both the hummus group (*p* < 0.001) and the falafel group (*p* < 0.001). Within the hummus group, traditional hummus received the highest median score (Me = 3.0), whereas the other hummus variants scored lower (Me = 2.0). In the falafel group, proprietary falafel and falafel with chickpeas and spelt achieved the highest median scores (Me = 3.0), while the remaining products had lower scores (Me = 2.0). The detailed results, including *p*-values and effect sizes, are presented in Table 11.

## 4. Discussion

In recent years, the trend of consuming plant-based products has been growing. Numerous scientific studies suggest that a well-balanced vegetarian diet contributes to the proper functioning of the body and provides health benefits compared to diets rich in animal-derived products [1].

Research indicates that plant-based diets significantly reduce the risk of developing cardiovascular diseases, hypertension, and type II diabetes. Additionally, the results of randomized clinical trials confirm that vegetarian diets contribute to weight reduction, which plays a crucial role in maintaining overall health [27].

Plant-based products have gained significant popularity, not only among vegetarian diet supporters but also within a broader group of consumers, becoming an essential element of daily nutrition [9]. The growing nutritional awareness of consumers significantly influences the development of healthy habits and dietary preferences when selecting appropriate food products [9].

The present study found that the majority of the respondents did not follow a vegetarian diet (236 surveyed individuals), with only a small group declaring adherence to a plant-based diet. According to the latest data published in the Consumer Insight Report (2024, CEOWORLD Magazine), vegetarians constitute 8.4% of the Polish population [28]. The study also indicated that only a small percentage of the respondents had followed a vegetarian diet for more than three years. Similar results were obtained in Goluch’s study [29], which found that one in four surveyed women declared adherence to a vegetarian diet for 6 to 10 years.

An improperly balanced vegetarian diet can significantly increase the risk of nutrient deficiencies and adverse effects. The present study found that the highest percentage of the respondents declared that they had no opinion regarding the most common drawbacks of a vegetarian diet. A significant portion of the respondents (70 surveyed individuals) indicated that the risk of vitamin and nutrient deficiencies was a key concern. These findings are supported by Bakaloudi’s review [2], which demonstrated that a vegetarian diet may contribute to deficiencies in certain micronutrients, such as vitamin B12, calcium, selenium, and zinc. Similarly, Śliwińska et al. [30] confirmed that 55% of vegetarians believe that a poorly balanced vegetarian diet can lead to vitamin and nutrient deficiencies.

In the study conducted by Bettinelli et al. [31], it was emphasized that a poorly balanced vegetarian diet in terms of nutrients may raise concerns about following this dietary pattern. However, the authors suggest the need to promote nutritional education on proper dietary practices and the health benefits of plant-based products, while reducing the consumption of animal-derived foods.

The study by Cader and Lesiów [9] found that one of the most common sources of information about vegetarian diets among respondents is the Internet (approximately 86% of the respondents). Similar results were obtained in the present study, where more than half of the respondents stated that they primarily obtain knowledge from websites, such as social media platforms and dietary forums (129 participants).

In the present study, based on the results concerning the perception of a vegetarian diet for children as healthy/appropriate, the respondents mostly considered it inappropriate (48.3%) and believed that it could lead to nutritional deficiencies (47.5%).

According to the American Dietetic Association (ADA) [32], plant-based diets, including vegan diets, when properly balanced, are safe, healthy, and nutritionally adequate for all age groups, starting from the earliest stages of life, including pregnancy, lactation, infancy, childhood, and adolescence.

In the present study, 90 respondents stated that they purchase vegetarian products in supermarkets, such as Biedronka, Lidl, and Auchan. This demonstrates that such products are widely available on the Polish market. The ease of purchasing vegetarian products in both physical stores and online shops indicates their broad accessibility and prevalence, as well as the continuous expansion of the distribution network in Poland. The respondents also indicated the significance of various quality indicators of food products that influence their purchasing decisions.

When asked which factors guide them when choosing food products, 122 respondents (47.5%) stated that price is a key determinant in their purchasing choices. It was also found that taste and product composition are very important quality indicators when making purchases. In contrast, brand, packaging size, and expiration date were considered by consumers as moderately important factors influencing their decisions. In the study conducted by Jankojć et al. [33], nearly 90% of the respondents declared that they pay special attention to the product composition before making a purchase. Similarly, in the study by Janowicz et al. [34], an analysis of consumer-preferred quality attributes of ready-to-eat food products showed that price is a key quality indicator. The second-most frequently mentioned factor by the respondents was product composition.

In the next phase of the study, the respondents (student group) conducted a consumer sensory evaluation of vegetarian products and their proprietary equivalents. The overall product evaluation included key quality indicators such as color, aroma, taste, consistency, and appearance, which were crucial in determining the overall quality rating of the product. The results obtained using the five-point scale method in this study showed that natural hummus received the highest ratings from the respondents. In contrast, among all the tested food products, the natural, gluten-free, organic hummus received the lowest score.

The natural hummus was rated the highest due to its balanced flavor, smooth texture, and absence of artificial additives, which aligns with consumer preferences for minimally processed foods. Prior studies have shown that hummus formulations with higher tahini content and natural ingredients tend to score better in sensory evaluations [35]. Additionally, Frankenfeld and Wallace [15] reported that the consumer acceptability of hummus is strongly influenced by the balance of chickpeas, tahini, and acidity from lemon juice. Our findings align with previous research indicating that consumers prefer traditional or homemade hummus over commercial variants due to a more authentic taste and texture [19].

In the study by Makhloufii and Yamani [36], the composition of plant-based spreads was evaluated by replacing chickpea seeds with dried green lentils, red lentils, dry white beans, and dried green peas. The sensory evaluation of the newly developed products was conducted using a nine-point hedonic scale. The sensory assessment of appearance, aroma, texture, taste, acidity, and overall acceptability of the samples showed statistically significant differences between the quality indicators and overall consumer acceptability (*p* ≤ 0.05). The consumer sensory evaluation of falafel showed that the participants rated the proprietary falafel the highest, whereas the vegan falafel received the lowest score from the respondents.

The next stage of the study involved assessing the consumer preferences among high school students using the ranking method. The evaluation focused on the selected quality attributes of the vegetarian products used in the study and their proprietary equivalents, specifically appearance and taste.

The results of the sensory evaluation using the ranking method showed that the participants who assessed the appearance of selected food products gave the highest scores to natural hummus and traditional hummus. In contrast, natural, gluten-free, organic hummus and proprietary hummus received lower scores. In the falafel group, the proprietary falafel received the highest score, while the other falafel products in the group were rated lower. The results of the sensory evaluation using the ranking method showed that the participants assessing the taste of the selected food products gave the highest scores for traditional hummus, while the other products in this group received significantly lower scores. In the falafel group, the proprietary falafel and falafel with chickpeas and spelt received the highest scores, whereas the traditional falafel, organic falafel, and vegan falafel were rated lower. The vast majority of the respondents (236 participants) declared that they do not follow a vegetarian diet, which may have significantly influenced the sensory evaluation results of the tested vegetarian products. The taste characteristics of vegetarian products may differ significantly from the preferences and eating habits of individuals who do not follow plant-based diets. The results of the study largely depend on the individual preferences of the participants.

### Strengths and Weaknesses of the Study

The study on vegetarian products stands out due to numerous strengths, emphasizing its scientific and practical value. One of its key advantages is the comprehensive sensory analysis, based on both the five-point scale method and the ranking method. This approach enabled a thorough evaluation of the quality of the tested products, significantly enriching the conclusions drawn from the research. An important aspect was also the inclusion of proprietary recipes for hummus and falafel, allowing for a direct comparison with their commercial counterparts. This feature enhanced the uniqueness of the study, providing valuable data for both food producers and consumers.

An additional advantage was the diversity of the study groups, which included students and high school students. This participant structure allowed for the collection of more varied opinions, enhancing the credibility of the results. Furthermore, the practical application of the study’s findings offers a real opportunity to improve the composition of food products and better tailor them to consumer expectations. The development process of proprietary recipes was conducted in a detailed and multi-stage manner, further increasing the reliability and quality of the obtained results.

Despite its many strengths, the study is not without limitations. One of them is the limited number of analyzed products, which makes it difficult to generalize the results to the broader market. Another limitation is the lack of data on long-term consumer preferences, preventing the ability to predict changes in their tastes and choices over time. Additionally, the study’s exclusive focus on the Polish market means that the findings may not be fully representative in the context of other countries.

The subjectivity of sensory evaluation presents another challenge, as even with structured methods, they remain susceptible to individual participant preferences. Additionally, the lack of detailed demographic data, such as socioeconomic status or lifestyle factors of the respondents, limits the ability to conduct a more precise analysis of how these variables influenced the obtained results.

In conclusion, the study provides valuable insights into the quality of vegetarian products and consumer preferences, while also holding significant potential for practical application. However, certain limitations highlight the need for further, more comprehensive analyses in the future to broaden the scope and enhance the representativeness of the collected data.

## 5. Conclusions

Our study showed that natural hummus and homemade falafel received the highest sensory scores, and taste was the key factor influencing consumer choices. Consumers preferred products with a simple composition and traditional recipe, which highlights the importance of the proper development of plant-based alternatives. The results suggest that nutritional education can play an important role in shaping conscious consumer choices, and further research should focus on optimizing the composition of plant-based products to increase their acceptance on the market.

The study provides numerous practical implications that can be applied in various areas of the food industry and consumer education. The findings may serve as a solid foundation for food manufacturers, enabling them to develop new, high-quality, and sensorially appealing vegetarian products that cater to the needs of potential consumers.

The data obtained in the study can also support educational campaigns promoting healthy and eco-friendly eating habits and be useful for dietitians, who can use these insights to recommend appropriate plant-based products for individuals following a vegetarian diet. Moreover, the results may inspire producers to support local initiatives and promote regional organic food producers, contributing to the strengthening of sustainable development.

An important aspect of the study is its potential to inspire innovative recipes and new applications for hummus and falafel, which could gain popularity as key components of consumers’ daily diets. In conclusion, the study provides valuable insights into consumer preferences and the quality of selected vegetarian products, which can significantly support the growth of the plant-based food market in Poland.

## Figures and Tables

**Table 1:** Composition of vegetarian products and their homemade equivalents used in the study.

Name of the Plant Product	Qualitative Composition
Pasta Hummus	
Natural hummus	cooked chickpeas bio (61%), water, sunflower oil bio, tahini bio (sesame paste bio), sea salt, apple cider vinegar bio, dried garlic bio
Hummus natural, organic, gluten-free, bio	soaked chickpeas (56%), water, sunflower oil, sesame paste (16%), sea salt, garlic, cumin, acidity regulator: lactic acid
Traditional hummus	chickpeas (57%), rapeseed oil, water, tahini sesame paste, lemon juice concentrate, salt, dried garlic, romaine cumin
Proprietary hummus ^1^	sesame seeds, olive oil, white chickpeas in brine, lemon juice, garlic, salt, roma cumin
Falafel	
Falafel traditional, bio	chickpeas (48%), onion, chickpea flour, courgette, mint (2%), corn grits, sea salt, sesame seeds (1%), parsley, lemon juice (1%), spices, sunflower oil
Vegan falafel	soaked chickpeas (50%), courgette, onion, breadcrumbs (wheat flour, yeast, salt), sunflower oil, parsley, cumin, salt, acidity regulator (potassium acetate, citric acid), coriander, garlic powder, black pepper
Falafel with chickpeas and spelt	chickpea flour (41%), heat-prepared spelt flour (39%), dried onion, dried parsley, spices (salt, garlic, chili, ginger, coriander, cinnamon, romaine cumin)
Proprietary falafel ^1^	white onion, garlic, parsley leaves, chickpeas in brine, romaine, salt, chili, spelt flour, baking powder

^1^ = homemade.

**Table 2:** Nutrient content of selected types of hummus. Own elaboration is based on product labels and food composition and nutritional value tables.

Names of Selected Hummus Pastes				
Nutritional Value per 100 g of Product	Natural Hummus	Hummus Natural. Organic. Gluten-Free. Bio	Traditional Hummus	Proprietary Hummus ^1^
Energy value [kJ/kcal]	1060/256	1210/289	782/189	1269/303.5
Fat [g]/ saturated fatty acids [g]	18.7/2.2	24.9/3.3	13.8/1.1	27.4/2.33
Carbohydrates [g]/ sugars [g]	10.0/1.7	5.9/1.2	7.5/0.6	11.6/2.1
Dietary fiber [g]	6.7	7.3	8.0	4.2
Protein [g]	8.5	7.5	4.7	5.2
Salt [g]	1.05	0.9	1.3	0.0

^1^ = homemade.

**Table 3:** Nutrient content of selected falafel types. Own elaboration is based on product labels and food composition and nutritional value tables.

Names of Selected Falafel Types				
Nutritional Value per 100 g of Product	Falafel Traditional. Bio	Vegan Falafel	Falafel with Chickpeas and Spelt	Proprietary Falafel ^1^
Energy value [kJ/kcal]	1052.0/252.0	845.0/203.0	1455.0/345.0	1755.8/419.4
Fat [g]/ saturated fatty acids [g]	11.0/1.3	11.2/1.3	3.9/0.7	19.7/1.9
Carbohydrates [g]/ sugars [g]	22.0/3.4	14.0/1.7	54.0/7.8	50.7/1.2
Dietary fiber [g]	12.0	10.1	13.0	7.6
Protein [g]	11.0	6.5	17.0	17.0
Salt [g]	1.9	1.5	3.0	0.5

^1^ = homemade.

**Table 4:** Sources of knowledge about a vegetarian diet (age of the respondents).

Sources of Knowledge	Age Groups			*p*-Value
	13–18 Years Old	18–24 Years Old	24–40 Years Old	
Websites (social networks, diet forums)	72 (55.4%)	49 (49.5%)	8 (32.0%)	*p* < 0.0001 *
Doctor’s/nutritionist’s opinion	12 (9.2%)	10 (10.1%)	4 (16.0%)	
Television (TV programs)	6 (4.6%)	0 (0.0%)	0 (0.0%)	
Books and scientific journals	10 (7.7%)	29 (29.3%)	15 (53.6%)	
I act on my own. I follow my intuition	30 (23.1%)	11 (11.1%)	1 (4.0%)	
Total [N]	130	99	28	

* = *p* < 0.05.

**Table 5:** Use of a vegetarian diet in a group of children and adolescents.

Use of a Vegetarian Diet	Level of Education			*p*-Value
	Primary (N = 131)	Secondary (N = 72)	Higher (N = 54)	
Yes	13 (9.9%)	14 (19.4%)	23 (42.6%)	*p* = 0.001 *
No	63 (48.1%)	39 (54.2%)	22 (40.7%)	
Do not know	55 (42.0%)	19 (26.4%)	9 (16.7%)	

* = *p* < 0.05.

**Table 6:** Evaluation of quality attributes relevant to consumers’ purchasing decisions.

Product Quality Features	Validity							
	Very Large		Large		Average		Small	
	[N]	[%]	[N]	[%]	[N]	[%]	[N]	[%]
Price	40	15.6	122	47.5	82	31.9	13	5.1
Product brand	10	3.9	70	27.2	111	43.2	66	25.7
Pack size	18	7.0	97	37.7	106	41.3	36	14.0
Product composition	109	42.4	91	35.4	44	17.1	13	5.1
Taste	186	72.4	65	25.3	6	2.3	0	0.0
Best before date	95	36.96	98	38.1	53	20.6	11	4.3

**Table 7:** Quality characteristics of selected vegetarian products.

Product Quality Features	[N]	Me	K_0.25_	K_0.75_	Xmin/Xmax
Price	257	3.0	2.0	3.0	1.0/4.0
Product brand	257	2.0	1.0	3.0	1.0/4.0
Pack size	257	2.0	2.0	3.0	1.0/4.0
Product composition	257	3.0	3.0	4.0	1.0/4.0
Taste	257	4.0	3.0	4.0	2.0/4.0
Best before date	257	3.0	3.0	4.0	1.0/4.0

Legend: [N]—sample size, Me—median symbol, K_0.25_—lower (first) quartile, K_0.75_—upper (third) quartile.

**Table 8:** Overall quality of hummus paste.

Product	Test Code	Quality Indicator	IC	[N]	$\bar{x}$	SD	Me	Xmin/Xmax
Natural hummus	435	Appearance	0.15	126	0.5	0.2	0.6	0.2/0.8
		Color	0.10	126	0.4	0.1	0.4	0.1/0.5
		Aroma	0.25	126	0.9	0.3	1.0	0.3/1.3
		Texture	0.15	126	0.6	0.1	0.6	0.2/0.8
		Taste	0.35	126	1.3	0.4	1.4	0.4/1.8
		Total Points	1	126	3.7	1.1	4.0	1.2/5.2
Hummus natural, organic, gluten-free, bio	300	Appearance	0.15	126	0.5	0.2	0.5	0.2/0.8
		Color	0.10	126	0.4	0.1	0.4	0.1/0.5
		Aroma	0.25	126	0.7	0.3	0.8	0.3/1.3
		Texture	0.15	126	0.5	0.2	0.5	0.2/0.8
		Taste	0.35	126	1.0	0.5	1.1	0.4/1.8
		Total Points	1	126	3.1	1.3	3.3	1.2/5.2
Traditional hummus	112	Appearance	0.15	126	0.6	0.2	0.6	0.2/0.8
		Color	0.10	126	0.4	0.1	0.4	0.1/0.5
		Aroma	0.25	126	0.8	0.3	0.8	0.3/1.3
		Texture	0.15	126	0.6	0.1	0.6	0.2/0.8
		Taste	0.35	126	1.3	0.3	1.4	0.4/1.8
		Total Points	1	126	3.7	1.0	3.8	1.2/5.2
Proprietary hummus ^1^	10	Appearance	0.15	126	0.4	0.2	0.5	0.2/0.8
		Color	0.10	126	0.4	0.1	0.4	0.1/0.5
		Aroma	0.25	126	0.9	0.3	1.0	0.3/1.3
		Texture	0.15	126	0.4	0.2	0.5	0.2/0.8
		Taste	0.35	126	1.1	0.5	1.1	0.4/1.8
		Total Points	1	126	3.2	1.3	3.5	1.2/5.2
*p*-value ε^2^		Appearance	0.017 * 0.03					
		Color	<0.001 * 0.07					
		Aroma	<0.001 * 0.19					
		Texture	<0.001 * 0.12					
		Taste	0.153 0.02					

Legend: IC—importance coefficient, $\bar{x}$—symbol for the arithmetic mean, SD—standard deviation, [N]—sample size, Me—median symbol, ε^2^—effect size, * *p* < 0.05, ^1^ = homemade.

**Table 9:** Overall quality of falafel.

Product	Test Code	Quality Indicator	IC	[N]	$\bar{x}$	SD	Me	Xmin/Xmax
Falafel traditional, bio	455	Appearance	0.15	126	0.5	0.1	0.5	0.3/0.8
		Color	0.10	126	0.4	0.1	0.4	0.1/0.5
		Aroma	0.25	126	0.8	0.3	0.8	0.3/1.3
		Texture	0.15	126	0.5	0.1	0.5	0.2/0.8
		Taste	0.35	126	1.2	0.4	1.4	0.4/1.8
		Total Points	1	126	3.4	1.0	3.6	1.3/5.2
Vegan falafel	222	Appearance	0.15	126	0.5	0.2	0.5	0.2/0.8
		Color	0.10	126	0.3	0.1	0.3	0.1/0.5
		Aroma	0.25	126	0.9	0.3	1.0	0.3/1.3
		Texture	0.15	126	0.5	0.2	0.5	0.2/0.8
		Taste	0.35	126	1.2	0.4	1.1	0.4/1.8
		Total Points	1	126	3.4	1.2	3.4	1.2/5.2
Falafel with chickpeas and spelt	60	Appearance	0.15	126	0.5	0.2	0.6	0.2/0.8
		Color	0.10	126	0.3	0.1	0.3	0.1/0.5
		Aroma	0.25	126	0.8	0.3	1.0	0.3/1.3
		Texture	0.15	126	0.5	0.2	0.5	0.2/0.8
		Taste	0.35	126	1.1	0.4	1.1	0.4/1.8
		Total Points	1	126	3.2	1.2	3.5	1.2/5.2
Proprietary falafel ^1^	100	Appearance	0.15	126	0.6	0.1	0.6	0.2/0.8
		Color	0.10	126	0.4	0.1	0.4	0.1/0.5
		Aroma	0.25	126	1.0	0.3	1.0	0.3/1.3
		Texture	0.15	126	0.6	0.1	0.8	0.2/0.8
		Taste	0.35	126	1.4	0.4	1.4	0.4/1.8
		Total Points	1	126	4.0	1.0	4.2	1.2/5.2
*p*-value ε^2^		Appearance	<0.001 * 0.23					
		Color	<0.001 * 0.06					
		Aroma	<0.001 * 0.20					
		Texture	<0.001 * 0.05					
		Taste	<0.001 * 0.05					

Legend: IC—importance coefficient, $\bar{x}$—symbol for the arithmetic mean, SD—standard deviation, [N]—sample size, Me—median symbol, ε^2^—effect size, * *p* < 0.05, ^1^ = homemade.

**Table 10:** Evaluation of the appearance of selected vegetarian products.

Product	Test Code	Quality Indicator	[N]	$\bar{x}$	SD	Me	Xmin/Xmax	*p*-Value ε^2^
Natural hummus	435	Appearance	131	2.4	1.1	3.0	1.0/4.0	<0.001 * 0.10
Hummus natural, organic, gluten-free, bio	300	Appearance	131	2.5	1.0	2.0	1.0/4.0	
Traditional hummus	112	Appearance	131	3.1	1.0	3.0	1.0/4.0	
Proprietary hummus ^1^	10	Appearance	131	2.1	1.1	2.0	1.0/4.0	
Falafel traditional, bio	455	Appearance	131	2.0	1.1	2.0	1.0/4.0	<0.001 * 0.15
Vegan falafel	222	Appearance	131	2.6	1.0	2.0	1.0/4.0	
Falafel with chickpeas and spelt	60	Appearance	131	2.4	1.1	2.0	1.0/4.0	
Proprietary falafel ^1^	100	Appearance	131	3.1	1.0	3.0	1.0/4.0	

Legend: $\bar{x}$—symbol for the arithmetic mean, SD—standard deviation, [N]—sample size, Me—median symbol, ε^2^—effect size, * *p* < 0.05, ^1^ = homemade.

**Table 11:** Taste evaluation of selected vegetarian products.

Product	Test Code	Quality Indicator	[N]	$\bar{x}$	SD	Me	Xmin ÷ Xmax	*p*-Value ε^2^
Natural hummus	435	Taste	131	2.3	1.1	2.0	1.0 ÷ 4.0	<0.001 * 0.04
Hummus natural, organic, gluten-free, bio	300	Taste	131	2.4	1.1	2.0	1.0 ÷ 4.0	
Traditional hummus	112	Taste	131	2.9	1.1	3.0	1.0 ÷ 4.0	
Proprietary hummus ^1^	10	Taste	131	2.4	1.2	2.0	1.0 ÷ 4.0	
Falafel traditional, bio	455	Taste	131	2.1	1.0	2.0	1.0 ÷ 4.0	<0.001 * 0.20
Vegan falafel	222	Taste	131	2.0	0.9	2.0	1.0 ÷ 4.0	
Falafel with chickpeas and spelt	60	Taste	131	2.8	1.1	3.0	1.0 ÷ 4.0	
Proprietary falafel ^1^	100	Taste	131	3.2	1.0	3.0	1.0 ÷ 4.0	

Legend: $\bar{x}$—symbol for the arithmetic mean, SD—standard deviation, [N]—sample size, Me—median symbol, ε^2^—effect size, * *p* < 0.05, ^1^ = homemade.

## Data Availability

The original contributions presented in this study are included in the article. Further inquiries can be directed to the corresponding author.

## Appendix A

### Appendix A.1. Preparation Method for Hummus Paste “Hummus with Homemade Tahini”

1. Weigh and place sesame seeds into a bowl.
2. Toast the sesame seeds in a dry, preheated pan until they turn golden brown.
3. Grind the toasted sesame seeds, add olive oil, and blend thoroughly to form a smooth tahini paste.
4. In a separate container, combine chickpeas, canned liquid (aquafaba), garlic, salt, cumin, and lemon juice.
5. Blend all ingredients until a smooth and creamy consistency is achieved.
6. Transfer the prepared hummus paste to another container for serving or storage.

### Appendix A.2. Preparation Method for “Herb Falafels”

1. Finely chop the prepared onion, garlic, and parsley.
2. Add canned chickpeas, cumin, chili, and salt to the chopped ingredients.
3. Blend all the ingredients using a blender until a smooth mixture is formed.
4. Incorporate flour and baking powder into the mixture.
5. Refrigerate the mixture for approximately 1 h to allow it to firm up.
6. Shape small patties from the chilled mixture, about 2 cm thick.
7. Heat oil in a non-stick pan.
8. Fry the falafels over a medium heat for 3–4 min on each side, until they turn golden brown.
9. Drain any excess oil from the fried falafels using a paper towel.

**Table A1:** Preparation of vegetarian products and their proprietary equivalents for sensory evaluation and sample size.

Product	Preparation of the Product for Testing	Sample Size
Pasta hummus	Storage of products in the refrigerator until serving.	Separation of samples weighing approximately 15 g.
Proprietary falafel	Frying the product for 5 min on each side in a small amount of oil. Cutting the products into small pieces (squares).	Separation of samples weighing approximately 30 g.
Falafel	The other falafels did not require any special preparation prior to administration for the study.	Separation of samples weighing approximately 30 g.

## References

[1] Mehta V. (2018). Vegetarian diet: A boon or bane for health?. J. Med. Res. Innov..

[2] Bakaloudi D.R., Halloran A., Rippin H.L., Oikonomidou A.C., Dardavesis T.I., Williams J., Wickramasinghe K., Breda J., Chourdakis M. (2021). Intake and adequacy of the vegan diet. A systematic review of the evidence. Clin. Nutr..

[3] Mocarska A. (2021). Postrzeganie wegańskiej oferty rynkowej przez konsumentów w Polsce w świetle badań ankietowych. J. Life Cycle Assess..

[4] Saari A.U., Herstatt C., Tiwari R., Dedehayir O., Mäkinen J.S. (2021). The vegan trend and the microfundations of institutional change: A commentatory on food producers’ sustainable innovation journeys in Europe. Trends Food Sci. Technol..

[5] Beck V., Ladwig B. (2020). Ethical Consumerism: Veganism. WIREs Clim. Change.

[6] Miguel I., Coelho A., Bairrada C.M. (2021). Modelling attitude towards consumption of vegan products. Sustainability.

[7] Hargreaves S.M., Rosenfeld D.L., Moreira A.V.B., Zandonadi R.P. (2023). Plant-based and vegetarian diets: An overview and definition of these dietary patterns. Eur. J. Nutr..

[8] Hargreaves S.M., Raposo A., Saraiva A., Zandonadi R.P. (2021). Vegetarian diet: An overview through the perspective of quality of life domains. Int. J. Environ. Res. Public Health.

[9] Cader P., Lesiów T. (2021). Weganizm i wegetarianizm jako diety we współczesnym społeczeństwie konsumpcyjnym. Nauk. Inżynierskie Technol..

[10] International Vegetarian Union (IVU) (2013). Definitions. https://ivu.org/definitions.html.

[11] The WHOQOL Group (1995). The World Health Organization quality of life assessment (WHOQOL): Position paper from the World Health Organization. Soc. Sci. Med..

[12] Skevington S.M., Lotfy M., O’Connell K.A., WHOQOL Group (2004). The World Health Organization’s WHOQOL-BREF quality of life assessment: Psychometric properties and results of the international field trial. A report from the WHOQOL group. Qual. Life Res..

[13] Kibil I., Gajewska D. (2022). Wege Dieta Roślinna w Praktyce.

[14] Brzozowska S.K., Tańska M., Sosna P. (2018). Porównanie cen i składów wybranych hummusów dostępnych na rynku. Koła Naukowe—Szkołą Twórczego Działania, Tom 4.

[15] Frankenfeld C.L., Wallance T.C. (2020). Dietary patterns and nutritional status in relation to consumption of chickpeas and hummus in the U.S. population. Appl. Sci..

[16] Bondyra-Wiśniewska B., Kaczorek M., Pacyna S., Wedziuk A., Nagel P., Pawluk I. (2021). Legumes Are Healthy! A Practical Nutritional Guide on Reducing Meat Consumption in Favor of Plant-Based Products.

[17] Reister E.J., Belote L.N., Leidy H.J. (2020). The benefits of including hummus and hummus ingredients into the American diet to promote diet quality and health: A comprehensive review. Nutrients.

[18] Kutepova I., Rehm C.D., Friend S.J. (2023). UK Chickpea Consumption Doubled from 2008/09-2018/19. Nutrients.

[19] Zdrojewicz Z., Majewski J., Pająk J., Majewski M. (2017). Hummus—trochę egzotyki na talerzu. Med. Rodzinna.

[20] (2024). Falafel “K-Take It Veggie” Label.

[21] (2024). Falafel with Chickpeas and Spelt Label.

[22] (2010). Sensory Analysis–General Guidelines for the Design of Sensory Analysis Laboratories.

[23] Baryłko-Piekielna N., Matuszewska I. (2009). Sensory Analysis of Food. Fundamentals, Methods, Applications.

[24] Messina V. (2014). Nutritional and health benefits of dried beans. Am. J. Clin. Nutr..

[25] Wanat-Kańtoch G., Białek-Dratwa A., Białek-822 Dratwa A., Grajek M. (2022). Alternative Diets in Nutritional Counseling. Alternative Nutrition Guide.

[26] Caldeira B., dos Santos P.A. (2024). Protein recommendations for vegetarians and vegans. Health Soc..

[27] Wang T., Masedunskas A., Willett W.C., Fontana L. (2023). Vegetarian and vegan diets: Benefits and drawbacks. Eur. Heart J..

[28] Wilson D. (2024). Revealed: Countries with the Most Vegetarians in the World. https://ceoworld.biz/2024/01/21/revealed-countries-with-the-most-vegetarians-in-the-world-2024/.

[29] Goluch Z., Izydorczyk M., Tomaszewska M. (2022). Frequency and Preferences for Consumption of Fortified Products by Women Following a Vegetarian Diet. Medicine and Health in the Modern World.

[30] Śliwińska A., Olszówka M., Pieszko M. (2014). Knowledge Assessment on Vegetarian Diets among the Tri-City Population. Sci. J. Gdynia Marit. Univ..

[31] Bettinelli M.E., Bezze E., Morasca L., Plevani L., Sorrentino G., Morniroli D., Giannì M.L., Mosca F. (2019). Knowledge of Health Professionals Regarding Vegetarian Diets from Pregnancy to Adolescence: An Observational Study. Nutrients.

[32] Craig W.J., Mangels A.R. (2009). Position of the American Dietetic Association: Vegetarian Diets. J. Am. Diet. Assoc..

[33] Jankojć A., Lesiów T., Biazik E. (2016). Quorn™ Meat Substitutes on the Polish Market. Part 2. Eng. Sci. Technol..

[34] Janowicz M., Ciurzyńska A., Zielińska M., Lenart A. (2018). “Convenient” Vegetable Ready Meals in Consumer Evaluation. Food Process. Technol. Adv..

[35] Wallace T.C., Murray R., Zelman K.M. (2016). The nutritional value and health benefits of chickpeas and hummus. Nutrients.

[36] Makhloufi L., Yamani M.I. (2024). A Study of Physical, Chemical, and Sensory Characteristics of Novel Legume Dips. Int. J. Food Sci..

